# Evaluation of the severe preeclampsia classification criterion for antiphospholipid syndrome in a study of 40 patients

**DOI:** 10.1186/s13075-021-02518-7

**Published:** 2021-05-04

**Authors:** Maddalena Larosa, Véronique Le Guern, Nathalie Morel, Mériem Belhocine, Amelia Ruffatti, Nicolas Martin Silva, Romain Paule, Luc Mouthon, Michel Dreyfus, Jean-Charles Piette, Odile Souchaud-Debouverie, Catherine Deneux-Tharaux, Gaelle Guettrot-Imbert, Vassilis Tsatsaris, Emmanuelle Pannier-Metzger, Anne Murarasu, Andrea Doria, Nathalie Costedoat-Chalumeau

**Affiliations:** 1 AP-HP, Cochin Hospital, Internal Medicine Department, Centre de référence maladies auto-immunes et systémiques rares d’Ile de France, Paris, France; 2 Division of Rheumatology, Department of Medicine-DIMED, University of Padova, Via Giustiniani 2, 35128 Padova, Italy; 3 Sacré-Coeur Hospital, Internal Medicine Department, University of Montréal, Montréal, Canada; 4 University Hospital Center of Caen, Department of Internal Medicine, Caen, France; 5 Université de Paris, 27 Rue du Faubourg Saint Jacques, 75014 Paris, France; 6 University Hospital Center of Caen, Gynecology and Obstetrics Department, Caen, France; 7 AP-HP, Pitié-Salpétrière University Hospital, Internal Medicine and Clinical Immunology Department, Centre de référence maladies auto-immunes et systémiques rares de l’Ile de France, Paris, France; 8 Poitiers University Hospital, Department of Medicine, Internal Medicine Department, Paris, France; 9 INSERM U 1153, Obstetrical, Perinatal and Paediatric Epidemiology (EPOPé research team), DHU Risks in Pregnancy, Université de Paris, Paris, France; 10 AP-HP, Cochin Broca Hôtel-Dieu Hospital, Port-Royal maternity, DHU Risk in Pregnancy, INSERM Unité 1139, Paris, France; 11 INSERM U 1153, Center for Epidemiology and Statistics Sorbonne Paris Cité (CRESS), Paris, France

**Keywords:** Antiphospholipid syndrome, Systemic lupus erythematosus, Thrombosis, Pregnancy, Severe preeclampsia, Classification criteria

## Abstract

**Background:**

The criteria for antiphospholipid syndrome (APS) include severe preeclampsia and/or placental insufficiency leading to preterm delivery before 34 weeks of gestation, but this APS manifestation has been rarely studied. Thus, we report a series of severe preeclampsia occurred in patients with APS.

**Methods:**

We retrospectively analysed data of women with APS (Sydney criteria) who experienced severe preeclampsia with delivery before 34 weeks’ gestation between 2000 and 2017 at five French internal medicine departments and one Italian rheumatology unit.

**Results:**

The 40 women had a mean age of 30.5 ± 4.6 years at their first episode of preeclampsia; 21 were nulligravid (52.5%), 12 (30%) had already been diagnosed with APS, and 21 (52.5%) had a triple-positive antiphospholipid (aPL) antibody test.

Preeclampsia occurred at a median gestational age of 25.5 weeks (IQR 23-29). It was associated with HELLP in 18 cases (45%), eclampsia in 6 (15%), placental abruption in 3 (7.5%), catastrophic APS in 3 (7.5%), and foetal and neonatal death in 11 and 15 cases. Overall, 14 (35%) children survived, born at a median gestational age of 31 weeks.

Among other APS criteria, 16 women (40%) experienced at least one thrombosis, 17 (42.5%) an intrauterine foetal death, and 19 (47.5%) at least one episode of HELLP during follow-up (median 5 years, IQR = 2-8). None had three or more consecutive miscarriages. Notably, 12 women (30%) had systemic lupus erythematosus.

**Conclusions:**

Severe preeclampsia led to high mortality in the offspring. Almost half of these women experienced other APS features, but not three consecutive miscarriages.

**Supplementary Information:**

The online version contains supplementary material available at 10.1186/s13075-021-02518-7.

## Background

Antiphospholipid syndrome (APS) is defined by a combination of arterial and/or venous thrombosis, pregnancy morbidity, and persistent antiphospholipid (aPL) antibodies (Abs), namely lupus anticoagulant (LAC), anticardiolipin (aCL), and anti-ß2 glycoprotein-1 antibodies (anti-ß2GP1) [1]. The 2006 APS classification criteria [2] include three obstetric manifestations: (1) at least three unexplained and consecutive pregnancy losses before the 10th weeks of gestation, (2) an unexplained intrauterine foetal death (IUFD) ≥ 10th week, and (3) a preterm birth ≤ the 34th week because of severe preeclampsia or other recognised features of placental insufficiency.

Although these obstetric criteria were established by expert consensus, little is known about preeclampsia in APS patients. To our knowledge, no series of APS patients with at least 3 pregnancy losses before the 10th week of gestation is currently available. We recently reported a series of 65 APS patients with foetal deaths [3] in which foetal death was often the inaugural sign of APS and also frequently associated with other APS criteria, in particular, preeclampsia and thromboses, while the ‘3 consecutive early miscarriages’ criterion was met in only one patient [3]. A series of 16 pregnancies complicated by Haemolysis, Elevated Liver Enzymes and Low Platelets (HELLP) syndrome in 15 women with APS, published in 2005, found HELLP syndrome was associated with preeclampsia/eclampsia [4]. This study did not, however, assess the other classification criteria.

Since no data are available from women with preeclampsia and APS, we retrospectively analysed severe preeclampsia in a series of women with APS. We also assessed in those with preeclampsia the prevalence of other classification criteria for APS and of systemic lupus erythematosus (SLE).

## Methods

### Patients

This retrospective study took place in five French hospital internal medicine departments and one Italian hospital rheumatology unit, where women who met the inclusion criteria were analysed. These inclusion criteria were (1) at least one episode of severe preeclampsia [5] before 34 weeks’ gestation, (2) in pregnancies observed between 2000 and 2017, and (3) in women who met the revised classification criteria for APS [2].

Preeclampsia was defined as the onset of hypertension (≥ 140/90 mmHg) with proteinuria ≥ 0.3 g/24 h, after 20 weeks’ gestation [5], and was defined as severe when accompanied by one or more of hypertension ≥ 160/110 mmHg, proteinuria ≥ 5 g/24 h, oliguria < 500 ml/24 h, impaired liver function, epigastric or right-upper quadrant pain, cerebral or visual disturbances, pulmonary oedema or cyanosis, and foetal growth restriction with thrombocytopenia [5]. Neonatal death was defined as death of a neonate within 28 days after birth.

When a patient had more than one episode of severe preeclampsia, we analysed the first episode that met the inclusion criteria. We included both primary APS and APS associated with SLE (defined according to the SLICC classification criteria) [6]. Thirteen women had also been included in our study of IUFD in APS patients, although for 7 of them we considered a different pregnancy [3].

### Methods

We retrospectively collected from the patients’ medical charts demographic, clinical, laboratory, and ultrasound data as well as treatments received during pregnancy. We considered that women had been treated during pregnancy if they were on low-dose aspirin (LDA) and/or low-molecular-weight heparin (LMWH) before the diagnosis of severe preeclampsia.

The antibody profile at the time of the APS diagnosis was recorded, including LAC, IgG and IgM aCL, and IgG and IgM ant-β2GPI. As recommended by the International Society of Thrombosis and Hemostasis, the presence of LAC was explored by activated partial thromboplastin time (APTT) and dilute Russell’s viper venom time (dRVVT) in most patients and occasionally with the dilute prothrombin time and kaolin clotting time [7]. The results of aCL were considered positive when they exceeded the 99th percentile of the laboratory’s control values or if the laboratory had none, when they exceeded 40 IgG or IgM phospholipid units (GPL or MPL). The upper reference limits supplied by the laboratory performing the test were used for the anti-ß_2_GPI antibodies. In accordance with the classification criteria [2], women were included only when they had at least two positive laboratory tests 12 weeks apart or more and within 5 years of the qualifying event. Because this is a retrospective study, antibodies could not be tested in a centralised laboratory.

### Statistical analysis

Categorical data are expressed by proportions. All continuous variables are presented as means and standard deviations (SD) if their distributions are parametric and otherwise as medians and interquartile ranges (IQR). Significance for the univariate analysis was set at 0.05. Statistical analyses used STATA v.13.1 software.

## Results

### Patient’s characteristics

The study included 40 women. Their demographic, clinical, and serological variables are summarised in Table 1. Their mean age at index preeclampsia episode was 30.5 ± 4.6 SD, and it occurred during their first pregnancy in 21 women (52.5%). Overall, 14 women (35%) had at least one thrombotic event before the index episode, with a median time of 7.5 years (IQR 3–12) between the thrombotic and obstetric events. Nine women (22.5%) had had obstetric manifestations of APS before the index episode.

**Table 1:** Characteristics of the index pregnancies complicated by severe preeclampsia in 40 women with APS

Patient’s characteristics	***N*** = 40 (%)
Previous thrombotic events	14 (35.0)
Previous obstetric manifestations of APS	9 (22.5)
Known APS	12 (30.0)
Associated SLE^a^	11 (27.5)
Primiparous	21 (52.5)
Mean age at index pregnancy ± SD	30.5 ± 4.6
**Antibody profile**	
IgG/IgM anti-cardiolipin antibodies	34 (85.0)
IgG/IgM anti-β2GPI antibodies	25 (62.5)
LAC	33 (82.5)
Triple-positive aPL antibody tests	21 (52.5)
**Treatment during index pregnancy, before onset of preeclampsia**	
Only LDA	4 (10.0)
Only LMWH	4 (10.0)
LDA + LMWH	15 (37.5)
Hydroxychloroquine	7 (17.5)
Glucocorticoids	8 (20.0)
Immunosuppressants	3 (7.5)
None	17 (42.5)
**Features of severe preeclampsia**	
Term at onset, weeks of gestation (median, IQR)	25.5 (23–29)
**Foetal outcome**	
IUFD	11 (27.5)
Neonatal death	15 (37.5)
Survival at 28 days after birth	14 (35.0)
Gestational age at birth of surviving children (*n* = 14)	31 (27–33)
Preterm delivery (< 37 weeks) among surviving children (*n* = 14)	14 (100.0)
IUGR among surviving children	2 (14.3)
**Associated maternal complications**	
HELLP syndrome	18 (45.0)
Eclampsia	6 (15.0)
Placental abruption	3 (7.5)
Catastrophic APS	3 (7.5)

*APS* antiphospholipid syndrome, *IQR* interquartile range, *SLE* systemic lupus erythematosus; ^a^Nine women had SLE at index pregnancy, whereas 2 patients developed SLE during this pregnancy. *Abs* antibodies, *aPL* antiphospholipid, *LAC* lupus anticoagulant, *LDA* low dose aspirin, *LMWH* low-molecular-weight heparin, *HELLP* haemolysis, elevated liver enzymes, low platelet, *IUGR* intrauterine growth restriction, *IUFD* intrauterine foetal death, *N* number, *SD* standard deviation

APS was diagnosed before the index episode of preeclampsia in 12 women (30%), with a median follow-up of 5 years (IQR 3–12) between APS diagnosis and this episode, while the remaining 28 (70%) were diagnosed with APS when the preeclampsia occurred. Tests for LAC antibodies were positive in 30 women (82.5%), and aPL antibodies triple-positive in 21 (52.5%).

### Previous pregnancies

Before the index episode of preeclampsia, 19 women (47.5%) had had a total of 45 pregnancies that resulted in 11 live births (24.4%, including two premature births with IUGR associated with HELLP syndrome in one and non-severe preeclampsia in another), 13 miscarriages (28.9%), 11 IUFD (24.4%), and 10 elective abortions (22.2%). Finally, three women (15.8%) had two consecutive miscarriages before the index preeclampsia episode, but none had a history of three consecutive miscarriages.

### Description of the index pregnancy with severe preeclampsia

For various reasons (known APS, positive aPL, previous obstetric complications), 23 (57.5%) women were receiving a treatment during the index preeclampsia episode: 4 by LDA, 4 by LMWH, and 15 by both LDA and LMWH. Seven patients were also under treatment with hydroxychloroquine (HCQ), eight with glucocorticoids and three with immunosuppressive drugs. The other 17 women (42.5%) were not receiving any treatment at the onset of the preeclampsia episode.

The median gestational age at the index episode was 25.5 weeks’ gestation (IQR 23-29). Maternal complications occurred in 21 women (52.5%), including HELLP syndrome in 18 (45%), eclampsia in 6 (15%), placental abruption in 3 (7.5%), and/or catastrophic APS (CAPS) in 3 (7.5%). Foetal complications were observed in all index pregnancies and included 11 IUFD (27.5%) and 29 preterm live births, 15 of whom (born at a median term of 24 weeks) died before day 28. The 14 premature surviving children were born at a median term of 31 weeks (IQR 27–33), 2 (14.3%) with IUGR.

Doppler ultrasound examinations at or after 22 weeks’ gestation were available for 38 women and reported as abnormal in 17 (44.7%). Abnormalities included bilateral uterine artery proto-diastolic notches (*n* = 10) or a unilateral notch (*n* = 5) and/or reverse or absent end-diastolic umbilical flow (*n* = 2).

### Subsequent pregnancies

After a median follow-up of 3.5 years (IQR 2–6) after the index preeclampsia episode, 26 (65%) women had at least one new pregnancy, with a total of 37 new pregnancies. Treatment was LDA in 37 pregnancies (100%), LMWH in 33 (89.2%), and HCQ in 16 (43.2%). The overall outcomes were live births in 33 pregnancies (89.2%), IUFD in 3 (8.1%), and one miscarriage (2.7%). No woman had 3 consecutive miscarriages. Of the 33 live births, 20 (60.6%) were uncomplicated, while 13 had at least one complication including preeclampsia (*n* = 8), IUGR (*n* = 5), HELLP syndrome (*n* = 4) and/or preterm delivery (*n* = 4). No eclampsia, CAPS, or placental abruptions were observed.

### Thrombotic events

By the end of the follow-up after the index preeclampsia episode (median 3.5 years, IQR 2–6), 16 women (40%) had had at least one thrombotic event. The first thrombosis occurred before or at the time of the index episode, except in two who had their first thrombosis, one after delivery and the other 12 years afterwards. The sites of thromboses were venous in 12 women, arterial in 6, and microthrombotic in 4. Four women experienced CAPS, 3 simultaneously with preeclampsia and one before the index case.

### Associated autoimmune diseases

At the end of follow-up after the index preeclampsia episode (median 3.5 years, IQR 2–6), 12 women had been diagnosed with SLE (30%), 9 (22.5%) before the index episode, 2 (5%) during that episode, and 1 (2.5%) 4 years after it. The live birth rate did not differ between women with SLE and APS and those with primary APS at the time of the index pregnancy (*P* = 0.60).

## Discussion

We describe the largest series of 40 women with severe preeclampsia and confirmed APS. The median time of onset of the severe preeclampsia was 25.5 weeks of gestation, which explains the poor prognosis of these pregnancies: 11 IUFDs, 15 neonatal deaths, and only 14 surviving premature children, born at a median term of 31 weeks. Maternal complications were also frequent: 18 HELLP syndrome, 6 eclampsia, 3 placental abruption, and 3 CAPS. No maternal death was observed.

Severe preeclampsia led to the diagnosis of APS in 70% of cases and occurred during their first pregnancy in half the women; this timing explains why the majority did not receive the recommended treatment for APS before the index episode [8]. The prognosis for subsequent pregnancies was much better, even though maternal and foetal complications still occurred. All women received LDA then, and 89.2% of patients received therapeutic or prophylactic doses of LMWH, as recommended by the EULAR recommendations [8, 9].

Tests for aPL antibodies showed that most women had positive LAC (82.5%) and more than half had triple-positive aPL tests (52.5%). These results are very similar reports [3, 10, 11] including our previous study of 65 APS patients with IUFD (72% were LAC-positive, and 35% triple-positive) [3]. In the PROMISSE study of 385 SLE patients, LAC was associated with adverse pregnancy outcomes (OR 8.32, 95% CI 3.95–19.26) [12], and it is not surprising that we found a high rate of LAC compared to that observed in unselected APS patients [13]. At the end of the follow-up period, 30% of the women had been diagnosed with SLE, a rate quite similar to other APS series [3, 14] where SLE occurred in 29 to 36% of cases.

Because the obstetric APS classification criteria have not been studied in detail, we examined the overlap between the different criteria. Our finding that over the entire follow-up (median 5 years, IQR 2–8), 17 women (42.5%) had at least one IUFD suggests a substantial overlap between severe preeclampsia and IUFD. Other features of placental insufficiency were also observed in 19 women (47.5%), who had at least one episode of HELLP syndrome, while IUGR was reported in 21. Moreover, 16 patients (40%) experienced at least one thrombotic event, most before the index pregnancy and 4 women experienced CAPS, concurrent with preeclampsia in 3 cases. By contrast, the APS criterion of ‘three consecutive miscarriages’ was not observed in any of our patients, results similar to those reported in our retrospective series of IUFD: among 65 women with APS and IUFD, only one had 3 consecutive miscarriages [3]. These results might be due to the various immunological pathways and phenotypes of APS, as Bramham et al. [15] proposed, suggesting that patients be subdivided into three groups: recurrent miscarriage, late foetal loss, or early delivery due to placental dysfunction before 32 weeks’ gestation and thrombotic APS [15]. While thrombosis and foetal loss seem to overlap, the apparent difference of the ‘recurrent miscarriage’ criterion calls into question the relevance of this specific form of APS.

Our study has limitations, first and foremost, its retrospective nature. A potential consequence of this is the possibility that not all obstetrics departments in our centres looked for APS in all patients with early preeclampsia, so that this diagnosis might have been missed in some women. In addition, some immunological features were not assessed and the obstetric care of these women with APS was not standardised, as shown by the fact that 4 further pregnancies in 3 patients were not treated with LMWH. Finally, we cannot exclude the possibility that patients might have failed to observe early miscarriages and thus not reported them. In any case, to our knowledge, despite its small number of patients, it is nonetheless the largest cohort focusing on severe PE in patients with APS, and it provides valuable information on APS phenotype in such patients.

## Conclusions

In conclusion, during the 40 index pregnancies, severe preeclampsia occurred early and both the in utero and neonatal mortality in offspring were high. APS was characterised by a strong antiphospholipid tests, and SLE was frequent. Among these 40 APS patients, almost half also experienced at least one episode of thrombosis, HELLP syndrome, and/or IUFD. However, no woman met the ‘three consecutive miscarriages’ classification criterion; this finding suggests that the underlying physiopathology of these two obstetric phenotypes might be different.

## Supplementary Information

**Additional file 1.**

## Data Availability

The datasets used and/or analysed during the current study are available from the corresponding author on reasonable request.
