# Supplementary material for: Evaluation of the severe preeclampsia classification criterion for antiphospholipid syndrome in a study of 40 patients
Source: Arthritis Res Ther. 2021 May 4;23:134. doi: 10.1186/s13075-021-02518-7 (PMC8094564; doi:10.1186/s13075-021-02518-7)
Supplement: Supplementary file 1 — Additional file 1. [file 13075_2021_2518_MOESM1_ESM.docx]

**SUPPLEMENTARY MATERIAL**

- Hemolysis, elevated liver enzymes, Low Platelets (HELLP) syndrome was defined as aspartate amino transferase>2 fold the normal, platelet count <100.000/μL, and lactate dehydrogenase >600 U/L [1];
- Premature birth was defined as a live birth before 37 WG;
- Intra-uterine growth retardation (IUGR) was defined as an estimated fetal weight less than the 10^th^ percentile for gestational age [2];
- Neonatal death was defined as a death within 28 days from birth.

**Legend to Supplementary Material:**

1. Sibai BM. Diagnosis, controversies, and management of the syndrome of hemolysis, elevated liver enzymes, and low platelet count. Obstet Gynecol. 2004;103:5(Pt 1):981-91.
2. ACOG Practice bulletin no. 134: fetal growth restriction. Obstet Gynecol. 2013 May;121(5):1122-1133. doi: 10.1097/01.AOG.0000429658.85846.f9.
